# A Novel *WRN* Frameshift Mutation Identified by Multiplex Genetic Testing in a Family with Multiple Cases of Cancer

**DOI:** 10.1371/journal.pone.0133020

**Published:** 2015-08-04

**Authors:** Liu Yang, Guosheng Wang, Xinyi Zhao, Song Ye, Peng Shen, Weilin Wang, Shusen Zheng

**Affiliations:** 1 Department of Medical Oncology, First Affiliated Hospital, School of Medicine, Zhejiang University, Hangzhou, Zhejiang, P. R. China; 2 Department of Medical Oncology, Zhejiang Provincial People's Hospital, Hangzhou, Zhejiang, P. R. China; 3 Department of Medical Oncology, Beilun Branch of First Affiliated Hospital, School of Medicine, Zhejiang University, Ningbo, Zhejiang, P. R. China; 4 Key Laboratory of Combined Multi-organ Transplantation, Ministry of Public Health Key Laboratory of Organ Transplantation, Hangzhou, Zhejiang, P. R. China; 5 Division of Hepatobiliary and Pancreatic Surgery, Department of Surgery, First Affiliated Hospital, Zhejiang University School of Medicine, Hangzhou, Zhejiang, P. R. China; Uppsala University, SWEDEN

## Abstract

Next-generation sequencing technology allows simultaneous analysis of multiple susceptibility genes for clinical cancer genetics. In this study, multiplex genetic testing was conducted in a Chinese family with multiple cases of cancer to determine the variations in cancer predisposition genes. The family comprises a mother and her five daughters, of whom the mother and the eldest daughter have cancer and the secondary daughter died of cancer. We conducted multiplex genetic testing of 90 cancer susceptibility genes using the peripheral blood DNA of the mother and all five daughters. *WRN* frameshift mutation is considered a potential pathogenic variation according to the guidelines of the American College of Medical Genetics. A novel *WRN* frameshift mutation (p.N1370Tfs*23) was identified in the three cancer patients and in the youngest unaffected daughter. Other rare non-synonymous germline mutations were also detected in *DICER* and *ELAC2*. Functional mutations in *WRN* cause Werner syndrome, a human autosomal recessive disease characterized by premature aging and associated with genetic instability and increased cancer risk. Our results suggest that the *WRN* frameshift mutation is important in the surveillance of other members of this family, especially the youngest daughter, but the pathogenicity of the novel *WRN* frameshift mutation needs to be investigated further. Given its extensive use in clinical genetic screening, multiplex genetic testing is a promising tool in clinical cancer surveillance.

## Introduction

Over the past 30 years, highly penetrant genes conferring cancer predisposition have been identified in cancer-prone families. These genes follow Mendelian inheritance patterns. Studies have successfully linked mutations with various conditions, for instance, *BRCA1* and *BRCA2* in hereditary breast–ovarian cancer syndrome, DNA mismatch repair genes in Lynch syndrome, *P53* in Li–Fraumeni syndrome, and *APC* in familial adenomatous polyposis [1–3]. Testing for germline mutations of highly penetrant cancer predisposition genes provides valuable genetic information regarding patients and their families and can be used in cancer surveillance and patient monitoring.

The technology of next-generation sequencing (NGS) enables whole-genome sequencing (WGS), whole-exome sequencing (WES), as well as targeted sequencing of specific regions of interest such as multiplex genetic testing to identify rare genomic variants. Multiplex genetic testing using NGS allows efficient and cost-effective screening of a panel of cancer susceptibility genes. Briefly, the target DNA fragments were enriched with a cancer gene panel and sequenced by NGS. NGS produces a large amount of sequence data for mapping, alignment, and filtering to obtain genetic variants. To define the pathogenicity of variants, we evaluated all the identified mutations according to the guidelines of the American College of Medical Genetics (ACMG) [4].

In this study, we analyzed three cancer patients from one family. The mother and second daughter had lung adenocarcinoma, and the eldest daughter has endometrial cancer. Multiplex genetic testing of 90 cancer predisposition genes revealed *WRN* frameshift mutations in the three patients. The test was also conducted on the other three daughters, revealing that the youngest daughter also has the same *WRN* frameshift mutation. The aforementioned results suggest that the *WRN* frameshift mutation could be of importance in cancer surveillance for the youngest daughter.

## Methods

### Ethics statement

Ethical approval for this study was granted by the Ethics Committees of the First Affiliated Hospital, School of Medicine, Zhejiang University. The patients and their families received genetic counseling. We obtained written informed consent from the patients enrolled in this study.

### Subjects and samples

We analyzed a family, which included a mother with lung cancer and her five daughters; the eldest daughter has endometrial cancer, and the second daughter died of lung cancer. We also compared the sequencing data of 300 unrelated healthy matched controls to exclude common single nucleotide variations [5]. Whole blood was collected from all six family members. Genomic DNA, extracted with standard methods, was used for multiplex genetic testing and validation by Sanger sequencing.

### Clinical investigation

Surgically resected endometrial cancer tissue and a biopsied sample obtained by percutaneous lung centesis were subjected to pathological assessment to establish histological diagnosis. The following parameters were studied: clinical and diagnostic data (age, sex, and clinical features), Doppler ultrasound results, and computerized tomography (CT) scans. Follow-up examinations were also carried out.

### DNA library preparation

Each DNA sample was quantified by agarose gel electrophoresis and NanoDrop spectrophotometry (Thermo Scientific, Waltham, MA, USA). Libraries were prepared using the standard Illumina protocol. In brief, 3 μg of genomic DNA was fragmented by nebulization. Fragmented DNA with single A overhangs were ligated at the 3ʹ end of Illumina adapters, and 350-400bp products were selected. The size-selected products were PCR amplified, and each sample was tagged with a unique index during the procedure. The final product was validated using the Agilent Bioanalyzer (Agilent Technologies, Santa Clara, CA, USA).

### Cancer gene panel enrichment and sequencing

The amplified DNA was captured with a germline sequencing panel containing 90 cancer susceptibility genes (S1 Table) based on MyGenostics GenCap Enrichment Technologies (MyGenostics, Baltimore, MD, USA). The gene list contained 75 genes downloaded from the Cancer Gene Census (www.sanger.ac.uk/genetics/CGP/Census/) and 15 extra genes, such as *AXIN2* and *BARD1*, which have been shown to be cancer risk genes [6–11]. Biotinylated probes were designed to tile along the exon regions and exon–intron boundaries of the target genes. The capture experiment was conducted according to the manufacturer’s protocol. In brief, 1 μg of sequencing library DNA was mixed with Buffer BL and GenCap 90 tumor gene panel probe (MyGenostics) and heated at 95°C for 7 min and then at 65°C for 2 min on a thermal cycler. Buffer HY (23 μL; MyGenostics), pre-heated at 65°C, was added to the mixture and then held at 65°C with the thermal cycler lid heat on for 22 h for hybridization. MyOne beads (50 μL; Life Technology, Carlsbad, CA, USA) were washed three times in 500 μL of 1× binding buffer and were resuspended in 80 μL of 1× binding buffer. Consequently, 64 μL of 2× binding buffer was added to the hybrid mixture, which was then transferred to a tube with 80 μL of MyOne beads. The mixture was rotated for 1 h on a rotary shaker. The beads were then washed once with WB1 buffer at room temperature for 15 min and three times with WB3 buffer at 65°C for 15 min. The bound DNA was eluted with Buffer Elute and amplified using the following program: 98°C for 30 s (1 cycle); 98°C for 25 s, 65°C for 30 s, and 72°C for 30 s (15 cycles); and 72°C for 5 min (1 cycle). The PCR product was purified using SPRI beads (Beckman Coulter Genomics, Danvers, MA, USA), according to the manufacturer’s protocol. The enrichment libraries were sequenced on an Illumina HiSeq 2000 sequencer for paired-end sequencing of 100-bp reads.

### Data analysis and variant interpretation

After HiSeq 2000 sequencing, high-quality reads were retrieved from raw reads by filtering out low-quality reads and adaptor sequences using the Solexa QA package and the cutadapt program (http://code.google.com/p/cutadapt/), respectively. SOAPaligner (http://soap.genomics.org.cn/soapsnp.html) was used to align the clean read sequences to the hg19 human reference genome. The sequencing results are summarized in Table 1.

**Table 1:** Summary of sequencing results.

Sample	Initial bases on target (bp)	Base covered on target (bp)	Coverage of target region (%)	Average sequencing depth on target	Fraction of target covered with at least 4× (%)	Fraction of target covered with at least 10× (%)	Fraction of target covered with at least 20× (%)
I:1	650764	637596	98.0%	550.12	97.0%	94.9%	92.2%
II:1	650764	637025	97.9%	537.06	96.9%	94.6%	92.7%
II:2	650764	635094	97.6%	485.28	96.4%	94.7%	93.5%
II:3	650764	645608	99.2%	597.49	97.8%	95.9%	93.5%
II:4	650764	645526	99.2%	648.38	97.8%	96.1%	93.9%
II:5	650764	646005	99.3%	272.08	97.3%	95.7%	93.4%

After the PCR duplicates were removed by Picard software (http://picard.sourceforge.net/), the SNPs were initially identified using the SOAPsnp program. The reads were subsequently realigned to the reference genome using BWA (http://bio-bwa.sourceforge.net/), and the InDels were identified using the GATK program (http://www.broadinstitute.org/gsa/wiki/index.php/Home_Page). The identified SNPs and InDels were annotated using the exome-assistant program (http://122.228.158.106/exomeassistant). MagicViewer was used to view the short-read alignment and validate the candidate SNPs and InDels. Variants were initially filtered if they appeared in the 1000 Genomes Project database, in the ESP6500 database with a minor allele frequency threshold of >0.05, in the dbSNP database, and in the 300 in-house Asian Genome database [5]. The remaining variants were further screened using the Catalogue of Somatic Mutations in Cancer (COSMIC) database.

To define the pathogenicity of the variants, we evaluated all the identified variants and InDels according to the ACMG guidelines [4]. Variants were defined as potentially pathogenic if they produced a truncating codon, an initiation codon, or a splice donor/acceptor effect, or if the effect on protein function related to disease phenotype is reported in the literature. Otherwise, the missense, silent, and intronic variants were defined as variants of uncertain significance (VUS). The VUS were further evaluated by three algorithms, namely, PROVEAN, SIFT, and PolyPhen-2, to predict the effects on protein function [12–15].

All sequencing data (FASTQ) were deposited in the SRA database of the National Center for Biotechnology Information (NCBI, http://www.ncbi.nlm.nih.gov/sra) with the following accession numbers: SRR1563024, SRR1563027, SRR1563036, SRR1563037, SRR1563039, and SRR1563041. Table 2 shows individual patients and their corresponding SRA accession numbers.

**Table 2:** Clinical presentations and identified germline mutations of each individual.

Individual	Sex	Age (y)	Age at onset (y)	Histology	Tumor stage	Mutant gene	MutCount/ Sequencing depth	Amino acid change and origin	SRA accession numbers	Hom/Het
I:1	F	83	82	Lung adenocarcinoma	Ⅳ	WRN	345/738	N1370Tfs*23, Mat	SRR1563024	Het
						DICER1	113/255	N1112D, Mat		Het
						ELAC2	135/255	Y83C, Mat		Het
Ⅱ:1	F	54	52	Endometrial cancer	Ⅱ	WRN	396/888	N1370Tfs*23, Mat	SRR1563027	Het
						ELAC2	132/255	Y83C, Mat		Het
Ⅱ:2	F	52	50	Lung adenocarcinoma	Ⅳ	WRN	304/723	N1370Tfs*23, Mat	SRR1563036	Het
						DICER1	107/208	N1112D, Mat		Het
						ELAC2	140/255	Y83C, Mat		Het
Ⅱ:3	F	50		No evidence of cancer		ELAC2	134/255	Y83C, Mat	SRR1563037	Het
Ⅱ:4	F	47		No evidence of cancer		ELAC2	131/255	Y83C, Mat	SRR1563039	Het
Ⅱ:5	F	45		No evidence of cancer		WRN	58/148	N1370Tfs*23, Mat	SRR1563041	Het
						ELAC2	46/85	Y83C, Mat		Het

F, female; Mat, maternal family; Hom/Het, homozygote/heterozygote; SRA, Sequence Read Archive.

### Primer design, PCR amplification, and Sanger sequencing

The poorly covered regions from NGS were amplified and verified by Sanger sequencing. The variants identified by multiplex genetic sequencing were also validated with Sanger sequencing. In brief, primers were designed using Primer3 software [16]. Primer sequences for validation are listed in S2 Table. Identified variants, including *WRN* (c.4108DelA), *DICER1* (c.A3334G), and *ELAC2* (c.A248G), were amplified in duplicate from genomic DNA of the six family members by using Hot FirePol DNA polymerase (Solis BioDyne, Tartu, Estonia). Sanger sequencing was performed using the Big Dye Terminator Cycle v1.1 Sequencing Kit (Applied Biosystems, Carlsbad, CA, USA) and ABI Prism 3130 Genetic Analyzer (Applied Biosystems).

## Results

### Clinical features

**Fig 1:**
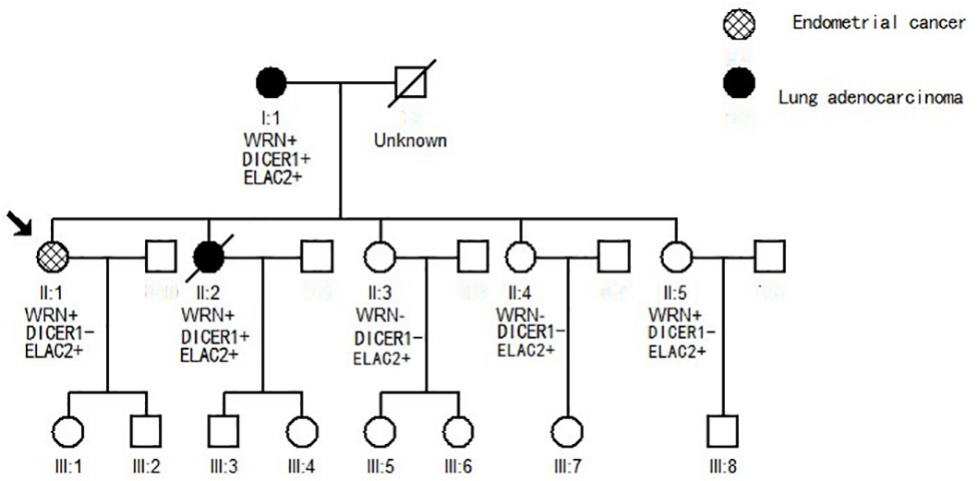
Pedigree and genotype–phenotype relationship of the family. The pedigree of individuals with cancer is represented by black circles (lung adenocarcinoma) and a gridded circle (endometrial cancer). A line through a symbol indicates that the person is deceased. The mutation status of *WRN*, *DICER1*, and *ELAC2* is presented in each individual who underwent multiplex genetic sequencing. A plus sign represents a mutant type, and a minus sign represents a wild type.

The proband is a 55-year-old woman (Fig 1, II:1), who is the oldest of five siblings. She experienced vaginal bleeding, and her menorrhea had stopped at the age of 48 years. Transvaginal ultrasonography showed an 8-mm pathologic mass on the right side of the uterine cavity. Magnetic resonance imaging (MRI) of the pelvic cavity suggested endometrial cancer, and a frozen section of the mass was consistent with endometrial cancer. Hysterectomy was performed, and the result of postoperative pathology confirmed the MRI results. The patient underwent radiation therapy after surgery. She exhibited no symptom of tumor recurrence or metastasis 6 months after surgery and continues with follow-up check-ups every 6 months.

The proband’s sister, a 50-year-old farmer and non-smoker (Fig 1, II:2), was in good health until she experienced a wracking cough, sputum production, and chest distress in 2012. She underwent a CT scan in the First Affiliated Hospital, School of Medicine, Zhejiang University. The scan revealed multiple masses of various sizes distributed over the whole lung and intensified diffuse lymphatic permeation. CT-guided percutaneous biopsy of the lung and histopathological examination demonstrated lung adenocarcinoma. The grade IV tumor was inoperable; thus, she accepted systematic therapy, which included chemotherapy and traditional Chinese drugs. The patient passed away in February 2014 at the age of 52 years.

The proband’s mother, an 80-year-old farmer and non-smoker (Fig 1, I:1), was in good health. In her physical examination, conducted in 2013, chest radiography revealed a 5-cm phyma in the lower right lung. No other symptoms were recorded. For further diagnosis and treatment, she underwent a comprehensive check-up, which included a chest and abdomen CT scan, head MRI, and bone emission CT scan at the First Affiliated Hospital. The results showed bone metastasis in the fourth and fifth vertebra lumbalis. CT-guided percutaneous biopsy of the lung was performed, and histopathological examination demonstrated lung adenocarcinoma. The patient was not a suitable candidate for surgical therapy. She gave up treatment because of advanced age and financial constraints. In the follow-up examination conducted 6 months after diagnosis, she remains alive, but her coughing and bone pain have worsened. The proband’s three younger sisters are still healthy. Their father had died from an accident 30 years ago.

### *WRN* frameshift mutation

The identified novel *WRN* frameshift mutation c.4108DelA (p.N1370Tfs*23) was shared among the mother (I:1), proband (II:1), second sister (II:2), and youngest unaffected sister (II:5); however, it is absent in the other two unaffected sisters (II:3 and II:4), as determined by multiplex genetic testing (Table 2). The *WRN* mutation resulted in a frameshift that introduced a stop codon 23 amino acids downstream of the mutation in exon 34, which is predicted to produce a truncated *WRN* protein. We further screened the *WRN* mutation in a cohort of 300 in-house entries in the Asian Genome database. The c.4108DelA (N1370Tfs*23) mutation was never found in healthy persons. The frameshift mutation of *WRN* is defined as potentially pathogenic, according to ACMG guidelines. No typical presentation of Werner syndrome was observed because the deleterious *WRN* mutations are heterozygous in the identified members of this family.

### Identification of other germline mutations

The missense mutation c.A3334G (p.N1112D) of *DICER1* was identified in both the lung cancer patients, namely, the affected mother (I:1) and the second daughter (II:2); however, it was absent in the other members of the family (Table 2). The *ELAC2* mutation c.A248G (p.Y83C) was found in all six family members (Table 2). *DICER1* and *ELAC2* mutations were defined as VUS according to ACMG guidelines. To further explore the effect of the previously mentioned germline mutations on protein function, we analyzed such mutations using the prediction tools PROVEAN, SIFT, and PolyPhen-2. *ELAC2* (p.Y83C) was predicted to be deleterious by PROVEAN, damaging by SIFT, and probably damaging by PolyPhen-2. *DICER1* (N1112D) was defined as neutral, tolerated, and benign by PROVEAN, SIFT, and PolyPhen-2, respectively. Table 3 presents the prediction scores.

**Table 3:** Annotation and functional prediction of germline mutations.

Gene	Accession number	Nucleotide change	Amino acid change	Mutation type	Mutation position	Hom/Het	PROVEAN prediction		SIFT prediction		PolyPhen-2 prediction		ACMG guidelines forvariant interpretation
							Score	Prediction (cutoff = -2.5)	Score	Prediction (cutoff = 0.05)	Score	Prediction	
WRN	NM_000553	4108DelA	N1370Tfs*23	Frameshift	Chr8:31024663	Het	-	-	-	-	-	-	Potential pathogenic
DICER1	NM_030621	A3334G	N1112D	Missense	Chr14:95570399	Het	-0.37	Neutral	0.218	Tolerated	1	Benign	VUS
ELAC2	NM_173717	A248G	Y83C	Missense	Chr17:12920436	Het	-7.96	Deleterious	0	Damaging	0	Probably damaging	VUS

Hom/Het, homozygote/heterozygote; V/T, variation reads/total reads; VUS, variants of uncertain significance; ACMG, American College of Medical Genetics.

### Validation of germline mutations of *WRN*, *DICER1*, and *ELAC2*

**Fig 2:**
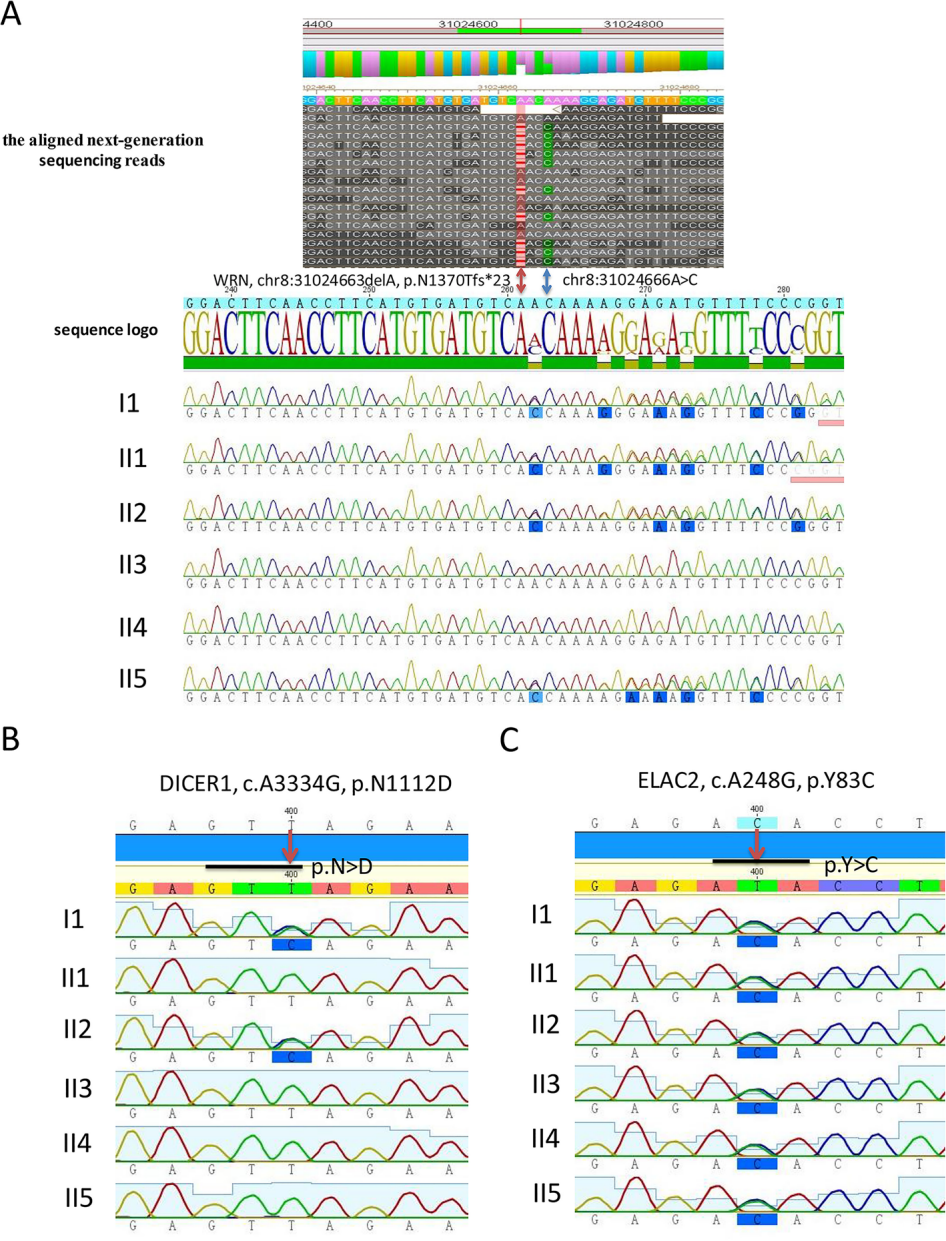
Sanger sequencing of germline mutations identified in *WRN*, *DICER1*, and *ELAC2*. (A) Sanger sequencing validation of *WRN* frameshift mutation in each individual. The aligned NGS data of *WRN* mutation from the mother (I:1). The *WRN* frameshift mutation presented a 1-bp deletion in chr8:31024663. The bases after A are shown in red, and the A→C point mutation in chr8:31024666 is shown in blue. The *WRN* frameshift mutation c.4108DelA (p.N1370Tfs*23) was validated by Sanger sequencing in the mother (I:1), the proband (II:1), the second daughter (II:2), and the youngest daughter (II:5); however, it was absent in the other two daughters (II:3 and II:4). (B) The *DICER1* missense mutation c.A3334G (p.N1112D) was validated by Sanger sequencing in the mother (I:1) and the second daughter (II:2), but it was absent in the other members of this family. (C) The *ELAC2* mutation c.A248G (p.Y83C) was validated by Sanger sequencing in all the members of this family.

We performed Sanger sequencing on the identified germline mutations in *WRN*, *DICER1*, and *ELAC2*. The results of Sanger sequencing were consistent with those of multiplex genetic testing (Fig 2).

## Discussion

Multiplex genetic testing combines selective genes capture and massive parallel sequencing. It efficiently facilitates simultaneous genetic analysis of a large number of candidate genes. Compared with the traditional stepwise gene-by-gene screening using Sanger sequencing, qPCR, or nucleotide mass spectrometry, the significant decrease in the cost of DNA sequencing makes multiplex genetic testing an efficient and economically advantageous approach. Multiplex genetic testing has been applied in clinical cancer genetics since 2012 [17, 18]. Recent published studies have robustly proven the clinical application of multiplex genetic testing in hereditary cancer risk assessment [19–21]. In this study, we identified a novel *WRN* frameshift mutation in three cancer patients and an unaffected family member using multiplex testing.

*WRN*, a member of the recombinase Q family of helicases, plays an important role in DNA repair function, including protecting the genome from abnormal recombination during chromosome segregation in mitosis and maintaining genomic stability [22]. Deficiencies in *WRN* cause Werner syndrome, which is a rare autosomal recessive disorder characterized by premature aging and cancer susceptibility [23–26]. All the mutations identified in *WRN* are predicted to truncate the *WRN* protein, with a loss of nuclear localization signal (NLS) in the C-terminal region (aa 1370–1375); moreover, the mutated *WRN* protein cannot be transported to the nucleus [27]. Impaired nuclear import is probably a major factor contributing to the molecular pathology of Werner syndrome. In this study, we identified a heterozygous frameshift mutation (p.N1370Tfs*23) in the NLS of the *WRN* C-terminal domain. In vitro analysis has confirmed that cell lines from *WRN* heterozygous individuals contain reduced *WRN* protein expression and reduced helicase activity [28]. *WRN* helicase activity is required to repair DNA inter-strand cross-links (ICLs) in cells, and *WRN* cooperates with *BRCA1* 452–1079 in cellular response to DNA ICLs [29]. Although the *WRN* heterozygote effect resulting from haploinsufficiency is supposed to be associated with *WRN* pathogenesis, this hypothesis needs to be further investigated.

*DICER1*, an important tumor suppressor gene, is an endoribonuclease that is important in generating microRNAs and short interfering RNAs [30]. Carriers of the *DICER1* germline mutation have been found to be at risk for pleiotropic tumor predisposition syndrome [31–33]. Interestingly, both the mother and the second daughter had lung cancer, which commonly presents with genetic variations in *DICER1*. The tendency suggests that *DICER1* may play an important role in lung carcinogenesis. Further functional studies can clarify the mechanism of *DICER1* in lung cancer. The identified *ELAC2* variant c.248A>G occurs within three nucleotides of a splice junction (intron1/exon2) and may affect gene splicing. The coding region of *ELAC2* consists of 826 amino acids of a metal dependency hydrolase, and this gene is the susceptibility gene for familial hereditary prostate cancer [34]. Other studies also found that the genotype of *ELAC2* is associated with a high risk of sporadic prostate cancer [35, 36].

Multiplex genetic testing of 90 cancer predisposition genes would produce genetic variants of uncertain clinical significance, which are referred to as incidental findings (IF). For *DICER1* and *ELAC2*, we cannot directly assess whether the identified missense mutations impair the function of the resulting protein. Although we used computational tools such as PROVEAN, SIFT, and PolyPhen-2 to predict whether the identified amino changes might be functionally significant, no direct functional assay was used. The ACMG recently published recommendations for reporting IF obtained from WGS and WES in clinical and research testing [37]. We used ACMG guidelines to define the mutations of *DICER1* and *ELAC2* as IF in this study. ACMG recommendations should be followed for multiplex genetic testing in clinical oncology. Informed consent is very important in NGS-based genetic testing used in clinical oncology. The American Society of Clinical Oncology (ASCO) has outlined the basic elements of informed consent for genetic testing used to assess cancer risk. In addition, information on data privacy, data security, testing laboratory licensure, availability of genetic counseling or cancer genetic risk assessment, and any possibility of future use of DNA samples should be incorporated into informed consent [38, 39].

Multiplex genetic testing successfully identified rare variations in cancer susceptibility genes in this study. However, penetrance estimates for such variants remain uncertain. Our study has certain limitations. The 90 cancer genes were selected from published literature; however, an optimal multiple gene panel for clinical oncology use remains to be defined. Without a direct functional assay, the clinical impact of identified rare variations cannot be predicted; therefore, it might not be appropriate to use the results to guide patient management. Furthermore, the outcome of the youngest daughter with the *WRN* frameshift mutation requires follow-up. Although more in-depth studies are warranted to guide clinical practice, our study shows an early indicator for the clinical correlation of multiplex genetic testing and highlights both benefits and drawbacks of large-scale genomic analysis in cancer-risk assessment.

## Supporting Information

S1 Table Cancer risk genes from the Cancer Gene Census. (XLSX)

S2 Table Primer sequences for validation by Sanger sequencing. (XLSX)
